# *Gnathovorax cabreirai*: a new early dinosaur and the origin and initial radiation of predatory dinosaurs

**DOI:** 10.7717/peerj.7963

**Published:** 2019-11-08

**Authors:** Cristian Pacheco, Rodrigo T. Müller, Max Langer, Flávio A. Pretto, Leonardo Kerber, Sérgio Dias da Silva

**Affiliations:** 1 Programa de Pós-Graduação em Biodiversidade Animal, Universidade Federal de Santa Maria, Santa Maria, RS, Brazil; 2 Centro de Apoio à Pesquisa Paleontológica da Quarta Colônia, Universidade Federal de Santa Maria, São João do Polêsine, RS, Brazil; 3 Laboratório de Paleontologia, Universidade de São Paulo, Ribeirão Preto, SP, Brazil

**Keywords:** Archosauria, Brazil, Carnian, Herrerasauridae, Paleobiology, Santa Maria Formation, Saurischia, Triassic, Candelária sequence, Dinosauria

## Abstract

Predatory dinosaurs were an important ecological component of terrestrial Mesozoic ecosystems. Though theropod dinosaurs carried this role during the Jurassic and Cretaceous Periods (and probably the post-Carnian portion of the Triassic), it is difficult to depict the Carnian scenario, due to the scarcity of fossils. Until now, knowledge on the earliest predatory dinosaurs mostly relies on herrerasaurids recorded in Carnian strata of South America. Phylogenetic investigations recovered the clade in different positions within Dinosauria, whereas fewer studies challenged its monophyly. Although herrerasaurid fossils are much better recorded in present-day Argentina than in Brazil, Argentinean strata so far yielded no fairly complete skeleton representing a single individual. Here, we describe *Gnathovorax cabreirai*, a new herrerasaurid based on an exquisite specimen found as part of a multitaxic association form southern Brazil. The type specimen comprises a complete and well-preserved articulated skeleton, preserved in close association (side by side) with rhynchosaur and cynodont remains. Given its superb state of preservation and completeness, the new specimen sheds light into poorly understood aspects of the herrerasaurid anatomy, including endocranial soft tissues. The specimen also reinforces the monophyletic status of the group, and provides clues on the ecomorphology of the early carnivorous dinosaurs. Indeed, an ecomorphological analysis employing dental traits indicates that herrerasaurids occupy a particular area in the morphospace of faunivorous dinosaurs, which partially overlaps the area occupied by post-Carnian theropods. This indicates that herrerasaurid dinosaurs preceded the ecological role that later would be occupied by large to medium-sized theropods.

## Introduction

Although much information has been already gathered about the largest predatory dinosaurs from the Jurassic and Cretaceous Periods, their early evolutionary history is still poorly understood, given the scarcity of fossils. Until now, knowledge concerning the some of the first top predatory dinosaurs mostly relies on herrerasaurids recorded in Carnian strata of South America. There are putative records of herrerasaurids from the mid-late Norian strata of Europe (Niedźwiedzki et al., 2014) and North America (Long & Murry, 1995; Hunt et al., 1998; Baron & Williams, 2018—but see Nesbitt, Irmis & Parker, 2007). Herrerasauridae unequivocally comprises three species (but see Baron & Williams, 2018): *Herrerasaurus ischigualastensis* (Reig, 1963), *Sanjuansaurus gordilloi* (Alcober & Martinez, 2010), both from the Ischigualasto Formation of Argentina, and *Staurikosaurus pricei* (Colbert, 1970) from the lower portion of the Santa Maria Formation of southern Brazil. Phylogenetic analyses recovered the clade in different positions within Dinosauria (Ezcurra & Novas, 2007; Baron, Norman & Barrett, 2017; Cabreira et al., 2016; Müller et al., 2018; Baron & Williams, 2018), whereas fewer studies challenged its monophyly (Sues, 1990; Nesbitt et al., 2017). Although herrerasaurid fossils are much better recorded in Argentina (Martinez et al., 2012) than in Brazil, Argentinean strata so far yielded no fairly complete herrerasaurid skeletons comprising a single individual (Novas, 1993). Here, we describe a new herrerasaurid based on an exquisitely preserved specimen found as part of a multitaxic association from southern Brazil. It corresponds to a nearly complete and well-preserved articulated skeleton, preserved in close association (side by side) with rhynchosaur and cynodont remains. Given its superb state of preservation and completeness, this specimen sheds light into poorly understood aspects of the Herrerasauridae anatomy (including endocranial soft tissues), reinforces the monophyletic status of the group, and provides clues on the ecomorphology of the early carnivorous dinosaurs.

## Materials & Methods

### Phylogenetic analysis

In order to infer its phylogenetic affinities and potential implications for understanding early dinosaur evolution, the new taxon was scored in a modified version of the data matrix of Müller et al. (2018), which is an updated version of that of Cabreira et al. (2016). In addition to the new herrerasaurid, eight new operational taxonomic units (OTUs) were included. These new OTUs and their respective sources are: the lagerpetid *Dromomeron gigas*, following the scores of Martínez et al. (2016) and Müller, Langer & Dias-da Silva (2018a); the ornithischians *Tianyulong confuciusi*, *Fruitadens haagarorum*, and *Echinodon becklesii*, following scores of Agnolín & Rozadilla (2018); the saurischian *Nhandumirim waldsangae*, following the scores of Marsola et al. (2018); and the sauropodomorphs *Bagualosaurus agudoensis*, *Unaysaurus tolentinoi*, and *Macrocollum itaquii*, following the scores of Pretto, Langer & Schultz (2018) and Müller, Langer & Dias-da Silva (2018b). Finally, the modifications in scores of *Pisanosaurus mertii* and *Silesaurus opolensis* performed by Agnolín & Rozadilla (2018) were also applied. The final data matrix includes 259 characters and 52 OTUs.

The data matrix was the subject of an equally weighted parsimony analysis in TNT v. 1.1 Goloboff, Farris & Nixon (2008). Characters 3, 4, 6, 11, 36, 60, 62, 64, 83, 115, 123, 139, 147, 148, 157, 160, 171, 173, 175, 178, 179, 182, 195, 200, 201, 202, 205, 216, 222, 240, and 248 were treated as ordered. *Euparkeria* was used to root the most parsimonious trees (MPTs), which were recovered with a ‘Traditional search’ (random addition sequence + tree bisection reconnection) with 1,000 replicates of Wagner trees (with random seed = 0), tree bisection-reconnection and branch swapping (holding ten trees per replicate).

### CT-scanning and three-dimensional reconstructions

The skull of CAPPA/UFSM 0009 was scanned with a Philips Brilliance 64-Slice CT Scanner (located at Santa Maria city), using 120 kV, 150.52 mAs. The analysis generated 1,002 slices with a 0.67 mm thickness, increment of 0.33 mm, and pixel size of 0.553 mm. Dragonfly 3.8 was used for rendering the three-dimensional volume of the skull. To access the endocranial information of CAPPA/UFSM 0009, its braincase was scanned with a µCT scan Skyscan™ 1173 at Laboratório de Sedimentologia e Petrologia of the Pontifícia Universidade Católica do Rio Grande do Sul (PUCRS), Porto Alegre, Brazil, using 130 kV and 61 µA. The scan resulted in 2,631 tomographic slices, with a voxel size of 29.98 µm. Before segmenting the regions of interest, slices without information were deleted, and the remaining files were cropped and binned (Balanoff et al., 2016) using the software ImageJ (Abràmoff, Magalhães & Ram, 2004). After these procedures, the sequence of tomographic slices was limited to 1,200, with a voxel size of 59.96 µm. The slices were imported in Avizo™ v. 8.1, the internal cavities were manually segmented, and 3D-models were generated (.stl format). The resulting 3D-models were coloured using Design Spark Mechanical 2.0 (the 3D models are in Pacheco et al. (2019) and the Supplementary File).

### Morphological disparity analysis

A morphological disparity analysis was performed in order to explore ecomorphological patterns among early dinosaurs and their relatives. We employed the dental characters of the phylogenetic taxon-character matrix (see Supplemental Information) and followed the parameters adopted by Müller & Garcia (2019), where an Euclidean distance matrix (EDMA) was calculated from the ecomorphological dataset using the software MATRIX (Wills, 1998). Then, a principal coordinate analysis (PCoA) was performed for the EDMA with the multivariate package GINKGO (Bouxin, 2005). The centroid of all operational taxonomic units (OTUs) was taken as the origin of multivariate axes, and the Cailliez method of negative eigenvalue correction was adopted. Finally, a bivariate graph with axes 1 and 2 of the PCoA was constructed using the software PAST (Hammer, Harper & Ryan, 2001).

### Terminology

This study employs veterinarian anatomical terms. Therefore, ‘cranial’/‘rostral’ and ‘caudal’ are used rather than the traditional or “Romerian” terms ‘anterior’ and ‘posterior’.

### Nomenclatural acts

The electronic version of this article in Portable Document Format (PDF) will represent a published work according to the International Commission on Zoological Nomenclature (ICZN), and hence the new names contained in the electronic version are effectively published under that Code from the electronic edition alone. This published work and the nomenclatural acts it contains have been registered in ZooBank, the online registration system for the ICZN. The ZooBank LSIDs (Life Science Identifiers) can be resolved and the associated information viewed through any standard web browser by appending the LSID to the prefix http://zoobank.org/. The LSID for this publication is: [urn:lsid:zoobank.org:pub:B555EB12-8CEA-4043-A652-4AFE6B02EC97]. The online version of this work is archived and available from the following digital repositories: PeerJ, PubMed Central and CLOCKSS.

## Results

### Systematic paleontology

**Table:** 

DINOSAURIA *Owen, 1842*
SAURISCHIA *Seeley, 1887*
HERRERASAURIDAE *Benedetto, 1973*
*Gnathovorax cabreirai* gen. et sp. nov.

**Holotype:** CAPPA/UFSM 0009 (Centro de Apoio à Pesquisa Paleontológica da Quarta Colônia/Universidade Federal de Santa Maria), an almost complete and partially articulated skeleton (lacking only part of the left shoulder girdle and part of the left forelimb).

**Figure 1:**
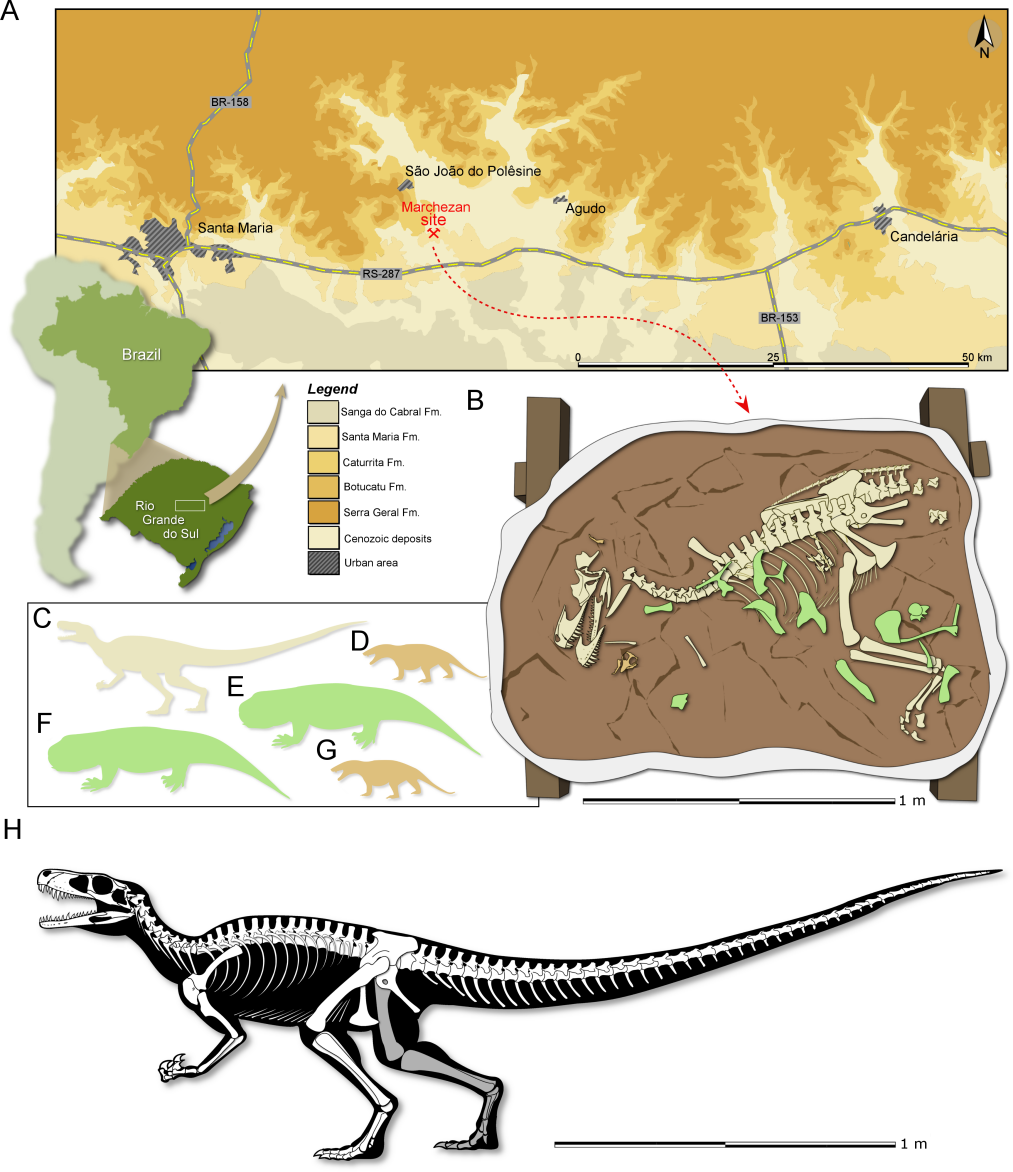
Study area and specimen. (A) Location map of the Marchezan site and the surface distribution of the geologic units in the area. (B) Schematic drawing of CAPPA/UFSM 0009 and associated specimens in the rock block before its final preparation. Silhouette of the associated individuals: (C) herrerasaurid; (D) cynodont/1; (E) rhynchosaur/1; (F) rhynchosaur/2 (collected near to the rock block); (F) cynodont/2. Silhouettes not to scale. (H) Reconstructed skeleton of *Gnathovorax cabreirai*.

**Locality and Horizon:** Marchezan site (29°37′52″S; 53°27′02″W), municipality of São João do Polêsine, Rio Grande do Sul, Brazil (Fig. 1A); Santa Maria Formation, Candelária Sequence, Paraná Basin (*sensu* Horn et al., 2014); stratigraphically correlated beds from a nearby site were dated as mid-Carnian (ca 233.23 ± 0.73), Late Triassic (Langer, Ramezani & Da-Rosa, 2018).

**Etymology:** The generic name is derived from the Greek *gnathos,* jaw, and the Latin *vorō* (“devour”) + −*āx* (“inclined to”). The specific epithet honours Dr. Sérgio Furtado Cabreira, the palaeontologist that found the specimen described here.

**Diagnosis:** *Gnathovorax cabreirai* differs from all other known herrerasaurids based on a unique combination of character states (*local autapomorphy): three premaxillary teeth*; presence of an additonal fenestra (=oval fenestra) between the maxilla and premaxilla contact, which is located dorsal to the subnarial fenestra, though markedly smaller than the subnarial fenestra*; two well defined laminae in the antorbital fossa of the maxilla, with a depression between them*; promaxillary fenestra absent*; slender ventral ramus of the lacrimal extending caudally almost until the midpoint of the orbit*; supraoccipital trapezoidal in caudal view; anterior portion of the basioccipital articulates with the parabasisphenoid through a V-shaped suture*, transverse processes of the last dorsal vertebra lacks contact with the ilium; distal end of the scapula craniocaudally expanded relative to the more proximal shaft; distally extended ente- and ectepicondyle in the humerus; preacetabular ala of the ilium with a pointed cranial tip*; pubis with a sinuous lateral margin in cranial/caudal views and a ventrally oriented shaft in lateral view; no depressed surface (=bevel) on the craniomedial region of the distal end of the pubis; presence of an opening on the obturator plate of the ischium*; proximal portion of the femur with lacks a caudomedial tuber; tibia equals to 90% of the femoral length; and three phalanges in pedal digit V*.

**Taphonomic remarks:** The skeleton of *Gnathovorax cabreirai* was preserved almost entirely articulated (Fig. 1B), lying on its right side in a mudstone layer, ridden with mudcracks and invertebrate bioturbation. Most of the missing elements are from its left side (mostly forelimb elements and ribs), indicating that the skeleton rested on the substract for an unknown amount of time before its final burial. The carcass as a whole shows no sign of transport, apart from the aforementioned elements that were carried away. Remains of small prozostrodont cynodonts, including semi-articulated specimens (Pacheco et al., 2018 and undescribed material) overlap the herrerasaurid skeleton, suggesting that the cynodonts were also not substantially transported, and may have roamed the environment surrounding the carcass. The mudstone that preserved both herrerasaurid and cynodonts was covered by a sandstone layer that also infilled the underlying mudcracks, indicating a moderate energy sedimentary episode. This sandstone preserved rhynchosaur bones, including limb elements, ribs, vertebrae and cranial material of at least two individuals. Some of the rhynchosaur elements overlapped the skeleton of *G. cabreirai*, which was also partially buried by the sandstone layer. The rhynchosaur bones were clearly deposited after the death of the herrerasaurid, but their disarticulation suggests that the bones were exposed for an extended time in the active taphonomic zone (or that the disarticulation occurred quickly, which seems less plausible), being later incorporated into this taphocoenosis probably via hydraulic transport. Yet, the absence of abrasion, rounded edges, and hydraulic selection, suggests that the rhynchosaur bones were not transported for a long time/distance. Therefore, it is likely that these three taxa (dinosaur, rhynchosaurs, and cynodonts) shared the same habitat in life (Fig. 1C).

**Figure 2:**
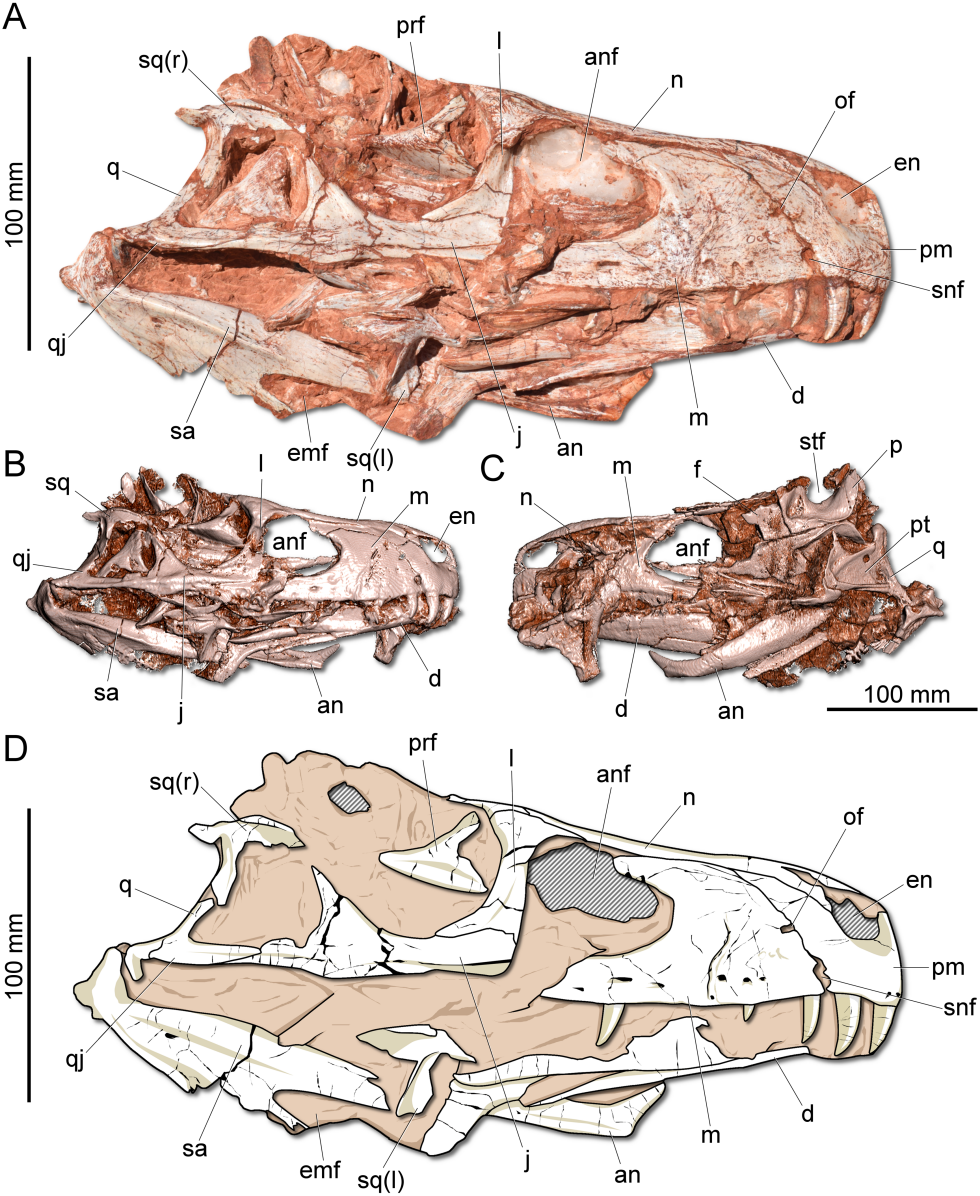
Photographs and reconstruction of the skull of CAPPA/UFSM 0009. (A) Right lateral view. (B) Three-dimensional rendering of the skull in right lateral view. (C) Three-dimensional rendering of the skull in left/dorsal lateral view. (D) Schematic drawing in right lateral view. an, angular; anf, antorbital fenestra; d, dentary; emf, external mandibular fenestra; en, external naris; j, jugal; l, lacrimal; m, maxilla; n, nasal; of, oval fenestra; p, parietal; pm, premaxilla; prf, prefrontal; pt, pterygoid; q, quadrate; qj, quadratojugal; sa, surangular; snf, subnarial foramen; sq, squamosal; stf, supratemporal fenestra.

**Figure 3:**
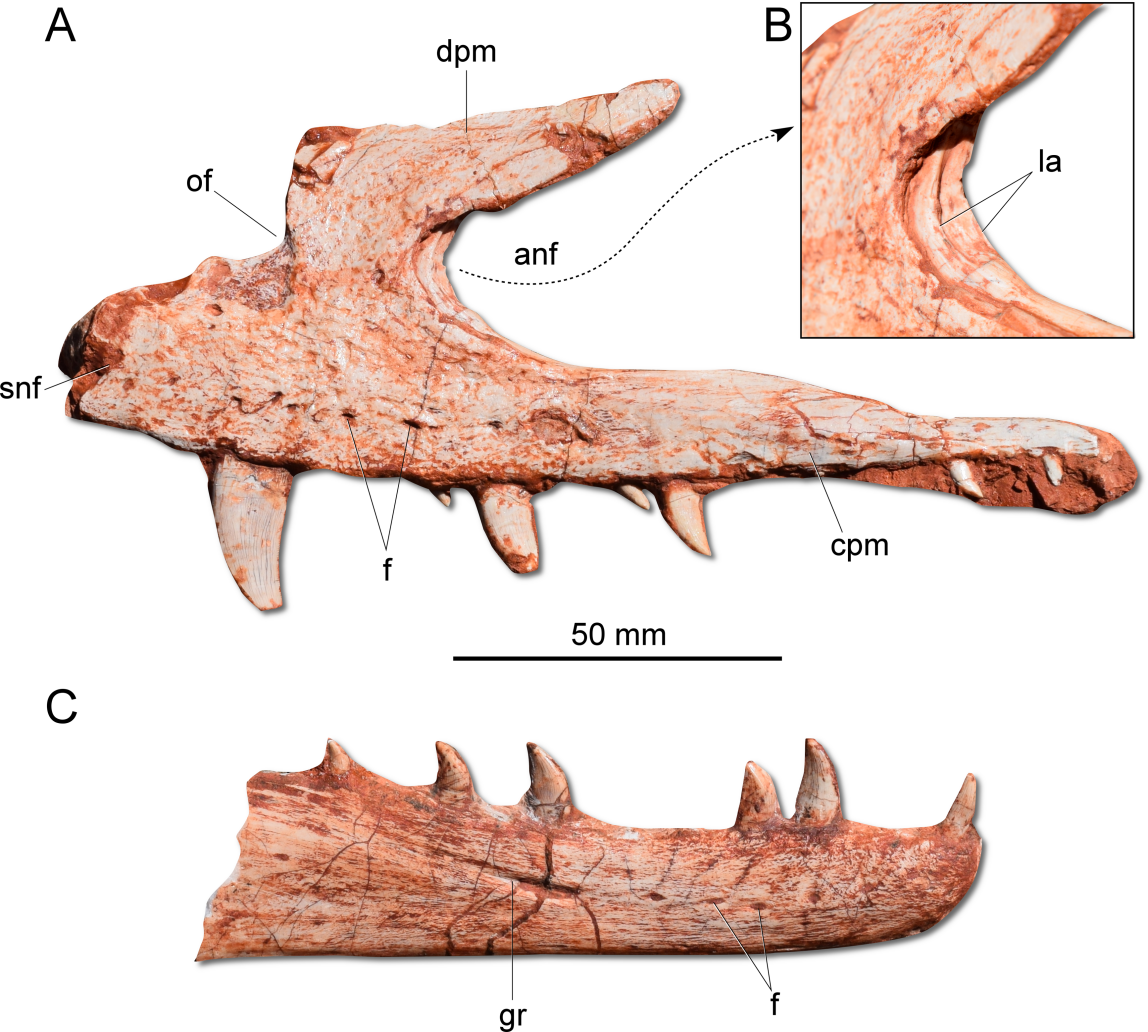
Photographs of selected skull bones of CAPPA/UFSM 0009. (A) Left maxilla in lateral view. (B) Magnification of the antorbital fossa of the left maxilla. (C) Right dentary in lateral view. anf, antorbital fenestra; cpm, caudal process of the maxilla; dpm, dorsal process of the maxilla; f, foramen; gr, groove; la, lamina; of, oval fenestra; snf, subnarial foramen.

**Description:** The skull of *Gnathovorax cabreirai* is almost entirely preserved (Fig. 2). Some of the bones from the left side are disarticulated, whereas the right side is better preserved and articulated. The body of the premaxilla is roughly quadrangular in lateral view and its caudodorsal process extends caudal of the caudal margin of the external naris, excluding the maxilla from its margin. The bone bears three tooth positions, whereas in *Herrerasaurus ischigualasensis* (Sereno & Novas, 1993) there are four. An oval fenestra is located between the premaxilla and maxilla, above the subnarial foramen and caudal to the external naris. That fenestra is smaller than the subnarial foramen, differing from *H. ischigualasensis* and possibly also *Sanjuansaurus gordilloi* (Alcober & Martinez, 2010). The maxilla presents a line of several foramina placed dorsal to the tooth row as in *H. ischigualasensis* and other saurischians. As in the aforementioned Argentinean herrerasaurids, the antorbital fossa is narrow, bordering the antorbital fenestra. *Gnathovorax cabreirai* lacks the promaxillary fenestra, which is seen in some other herrerasaurids, unaysaurid sauropodomorphs, *Eodromaeus murphi*, and most latter theropods (Sereno & Novas, 1993; Alcober & Martinez, 2010; Martinez et al., 2011; Müller, Langer & Dias-da Silva, 2018b). As in many other dinosaurs, an invagination is present between the maxillary ascending process and the lateral surface of the antorbital fossa. However, the new taxon uniquely shows a second invagination, forming two laminae in the antorbital fossa (Figs. 3A–3B). The ventral (=jugal) ramus of the lacrimal presents a slender caudal projection that extends along the ventral margin of the orbit (Fig. 1A), almost reaching its midpoint, as in *Daemonosaurus chauliodus*. This condition differs from that of *H. ischigualastensis*, in which the rostral lamina of the jugal is overlapped by a short ventral process of the lacrimal. As in many saurischians, the jugal has a prominent longitudinal ridge just dorsal to its ventral margin. In addition, distinct from early sauropodomorphs, the rostral tip of the jugal is dorsoventrally expanded. The infratemporal fenestra is trapezoidal in lateral view, with the dorsal half rostrocaudally shorter than the ventral, as found in *H. ischigualastensis*. The quadratojugal is partially fused to the quadrate. The ventral process of the squamosal is rostrocaudally broad, distinct from the strap-like shape of early sauropodomorphs, such as *Eoraptor lunensis* (Sereno, Martínez & Alcober, 2013), *Buriolestes schultzi* (Müller et al., 2018), and *Saturnalia tupiniquim* (Bronzati, Müller & Langer, 2019). On the dorsolateral surface of the bone, there is a marked slot for the postorbital, which is mediodorsally bounded by a ridge. The posterior process of the squamosal is tongue-like and proportionally longer than in *H. ischigualastensis.* The new taxon has a broad supratemporal fossa (Fig. 2C), which invades a significant portion of the caudal half of the frontal. Distinct from other non-herrerasaurid dinosaurs from Carnian (Cabreira et al., 2011; Martinez et al., 2011; Sereno, Martínez & Alcober, 2013), *G. cabreirai* lacks any palatal teeth.

**Figure 4:**
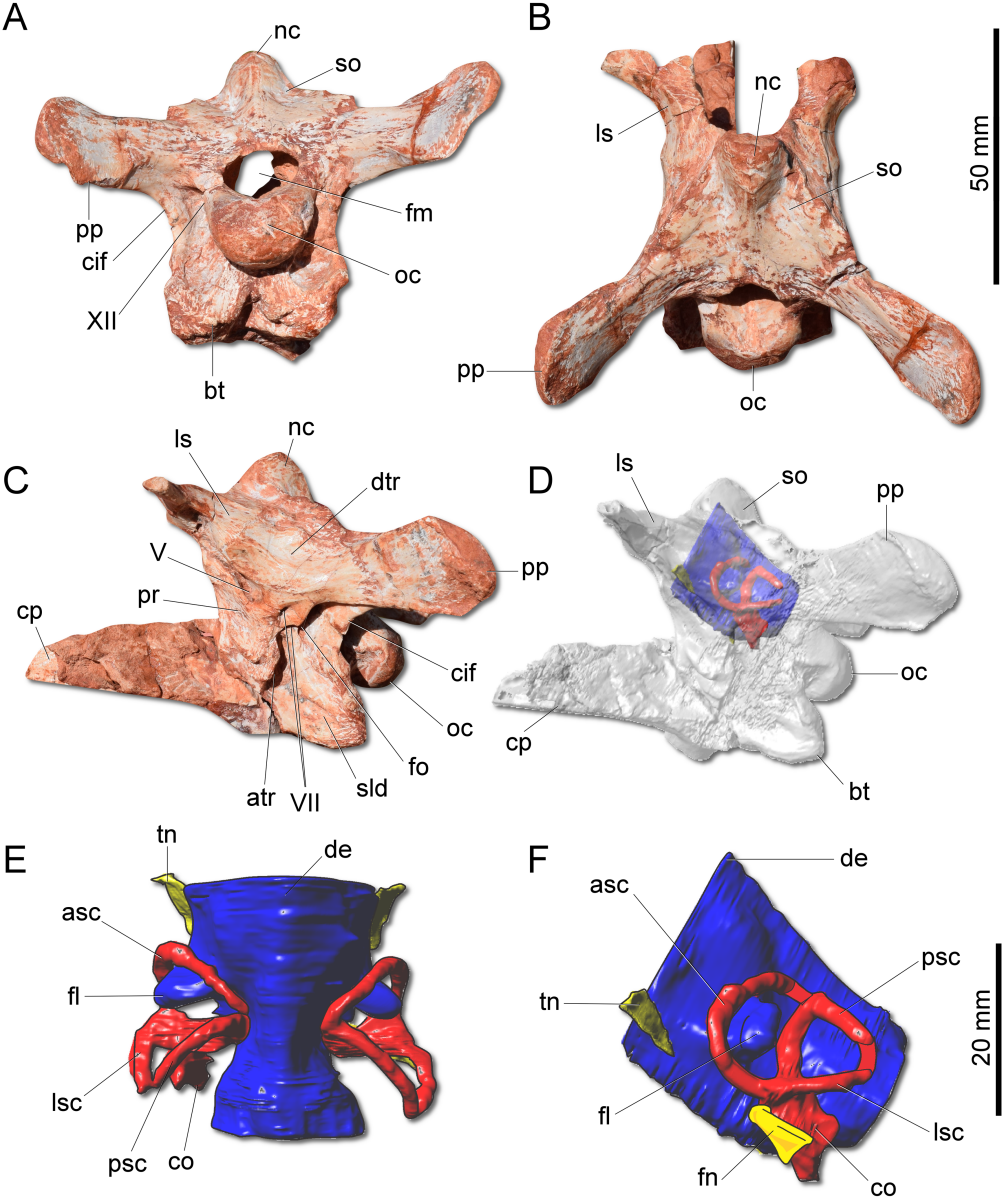
Photographs and reconstruction of the braincase and endocast of CAPPA/UFSM 0009. (A) Braincase in caudal view. (B) Braincase in dorsal view. (C) Braincase in left lateral view. (D) Three-dimensional rendering of the neurocranium in left lateral view with the endocast highlighted. (E) Endocasts of the brain, inner ear, and cranial nerves in dorsal view. (F) Endocasts of the brain, inner ear, and cranial nerves in left lateral view. asc, anterior semicircular canal; atr, anterior tympanic recess; bt, basal tubera; co, cochleae; cif, crista interfenestralis; cp, cultriform process; de, dorsal expansion; dtr, dosal tympanic recess; fl, floccular fossae lobe; fm, foramen magnum; fn, facil nerve; fo, fenestra ovalis; ls, laterosphenoid; lsc, lateral semicircular canal; nc, nuchal crest; oc, occipital condyle; pp, paraoccipital process; pr, prootic; psc, posterior semicircular canal; sld, semilunar depression; so supraoccipital; tn, trigeminal nerve; V, notch of the trigeminal nerve; VII, foramen for the facial nerve; XII, foramen for the hypoglossal nerve.

The supraoccipital of *G. cabreirai* is trapezoidal in caudal view (Fig. 4A), differently from the triangular bone of *H. ischigualastensis*. The bone bears a tall nuchal crest that is lateromedially compressed at its caudal portion and lacks any dorsal excavation for the vena occipitalis externa. The distal portion of the paraoccipital process is dorsoventrally expanded and the entire process is proportionally longer than in *H. ischigualastensis*. The laterosphenoid has two notches at its ventromedial margin. The rostral one corresponds to the medial margin of the foramen for the trochlear nerve (IV), whereas the caudal one corresponds to the medial margin for the foramen for the oculomotor nerve (III). In addition, the ventral contact of the laterosphenoid with the prootic bounds the entrance for the trigeminal nerve (V). Caudal to the notch for the trigeminal nerve, the prootic also bears a pair of foramina for the facial nerve (VII) (Fig. 4C). The floor of the endocranial cavity is smooth, lacking a longitudinal crest (=*eminentia medullaris*). The occipital condyle of *H. ischigualastensis* (PVSJ 407) is far more gracile compared to *G. cabreirai*. The anterior portion of the basioccipital articulates with the parabasisphenoid through a V-shaped suture, contrasting with the U-shaped suture of *H. ischigualastensis*. The parabasisphenoid presents a weak recess on its ventral surface.

The hindbrain endocast (medulla oblongata and the cerebellum) shows a constriction at the level of the inner ear and, on its dorsal surface, there is a longitudinal eminence (Figs. 4E–4F). There is a well-developed Floccular Fossa Lobe of the cerebellum (FFL) (see Bronzati et al., 2017), which forms a caudolaterally oriented protuberance, passing through the anterior semicircular canal. In the inner ear, the anterior semicircular canal (ASC) is rounded and more developed than the posterior (PSC) and lateral (LSC) and taller than the PSC. In dorsal view, the angle between ASC and PSC is 78°; in lateral view, the angle between PSC and LSC is around 60°, and 84 between LSC and ASC in anterior view. In lateral view, the crus commune is oriented 70° in relation to LSC and slightly curved caudally. The cochlear canal is ventrally projected, tapering in that direction.

The dentary is a long and robust bone with several vascular foramina along its length, where the last one lies on a longitudinal groove (Fig. 3C). The dentary symphysis is short and limited to the rostral portion of the bone. Contrasting with sauropodomorphs (Nesbitt, 2011; Cabreira et al., 2016; Bronzati, Müller & Langer, 2019), the dorsal surface of the rostral tip of the dentary does not deflect ventrally. Moreover, there is no edentulous gap between the rostral extremity of the dentary and the first tooth. Apparently, *G. cabreirai* possesses an intramandibular sliding joint similar to that inferred for *H. ischigualastensis* and *St. pricei*, which encompasses the dentary/surangular contact, dorsal to the external mandibular fenestra, and the splenial/angular articulation, ventral to that aperture. However, as both mandibular rami are broken in the area of these articulations, it is difficult to properly access their anatomy.

The dental formula of *G. cabreirai* is three premaxillary, 19 maxillary, and about to 14 dentary teeth. All tooth crowns are blade like, caudally curved and labiolingually compressed. The premaxillay and dentary teeth lack serrations in their mesial margins. However, in the distal margin there are small serrations that form a right angle with the main axis of the tooth. In the maxillary teeth the serration occur in both margins.

**Figure 5:**
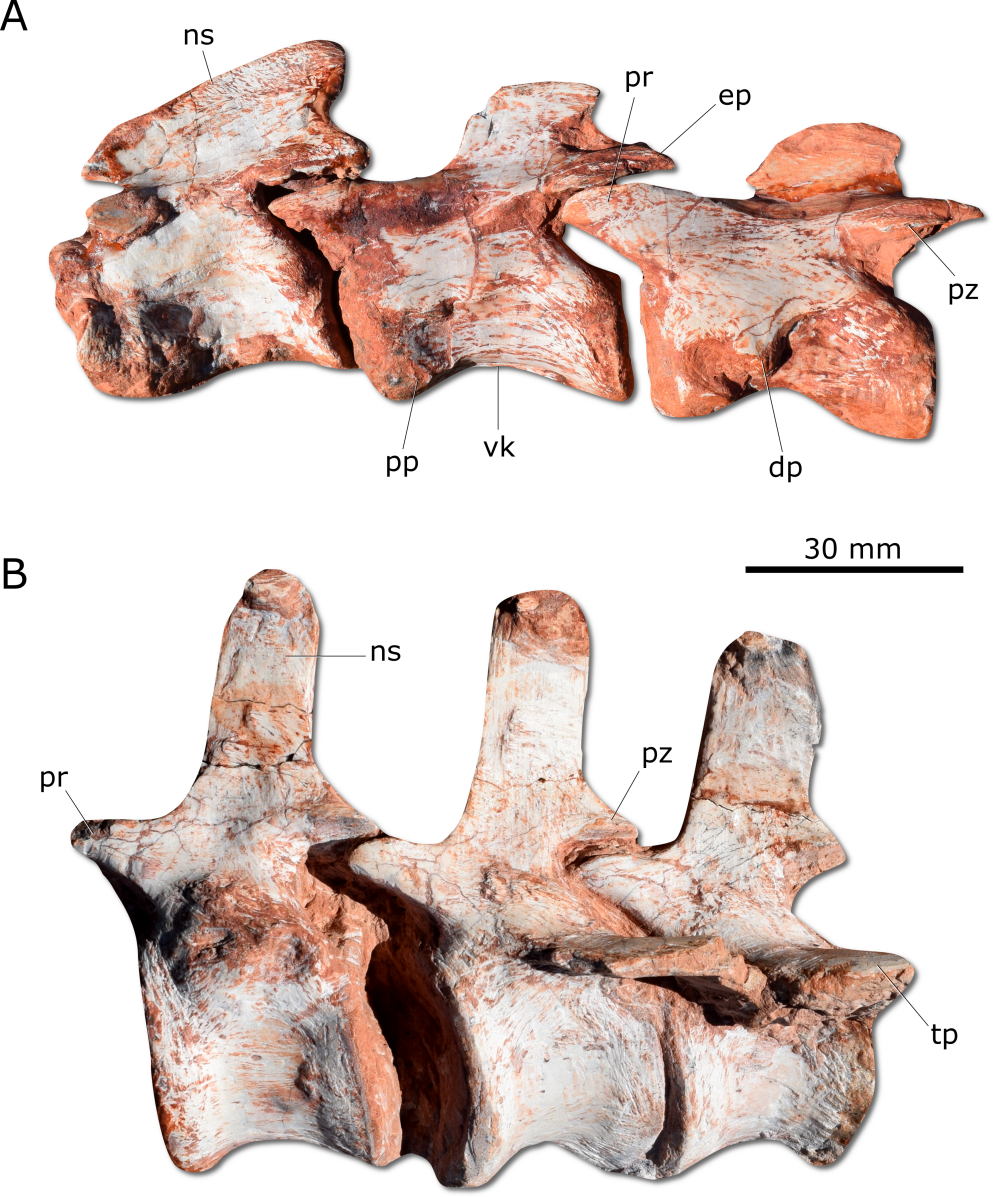
Photographs of selected axial elements of CAPPA/UFSM 0009. (A) second cervical vertebra (axis) to fourth cervical vertebra in left lateral view. (B) Proximal caudal vertebrae in left lateral view. dp, diapophysis; ep, epipophysis; ns, neural spine; pp, parapophysis; pr, prezygapophysis; pz, postzygapophysis; tp, tranverse process; vk, ventral keel.

*Gnathovorax cabreirai* preserves the complete cervical-dorsal series, with nine partially articulated cervical and 16 articulated dorsal vertebrae. Unlike most sauropodomorphs (Müller, Langer & Dias-da Silva, 2018a; Müller, Langer & Dias-da Silva, 2018b), the neck of the new taxon is short, with vertebral centra no more than 2.5 times longer than tall (Fig. 5A). There is a longitudinal keel on the ventral surface of the centrum of each cervical vertebra, as also seen in other herrerasaurids. *Gnathovorax cabreirai* resembles *San. gordilloi* regarding the presence of a long and caudolaterally directed transverse process in the caudal cervical vertebrae, which are considerably shorter in *H. ischigualastensis* and *St. pricei*. In the first dorsal vertebra, the ventral keel is markedly reduced if compared with that of the first cervical element. Also, the dorsal transverse processes are more elongated and laterally oriented, differing from those of the last cervical element, which are short and caudoventrally oriented. In addition, the first dorsal vertebra is 30% shorter than the last cervical one, as in *San. gordilloi.* The neural spines of the dorsal vertebrae are “H” shaped in cross-section, resembling the typical herrerasaurid condition. Unlike *San. gordilloi*, the new species lacks any cranial or caudal processes in the distal end of the neural spine. Nonetheless, some of the dorsal vertebrae show slight lateral expansions, forming a lateral expansion at the dorsal margin. The sacrum is composed of the two primordial sacral vertebrae, but a dorsal element seems to be positioned between the ilia. Its transverse processes are slender and project craniolaterally, almost reaching, but not contacting the ilia. The proximal (=cranial) caudal vertebrae (Fig. 5B) have tall centra (taller than the neural arches) and zygapophyses located far from the transverse process. The transverse processes are semi-circular in section, as in *H. ischigualastensis* and *St. pricei*, and not flattened as in *San. gordilloi*. As in other herrerasaurids, the neural spines of those vertebrae project vertically (Novas, 1993; Bittencourt & Kellner, 2009; Alcober & Martinez, 2010). In the distal caudal vertebrae, as in *H. ischigualastensis*, the prezygapophyses occupy a lower position, and gradually become longer, overlapping the distal half of the preceding vertebra.

**Figure 6:**
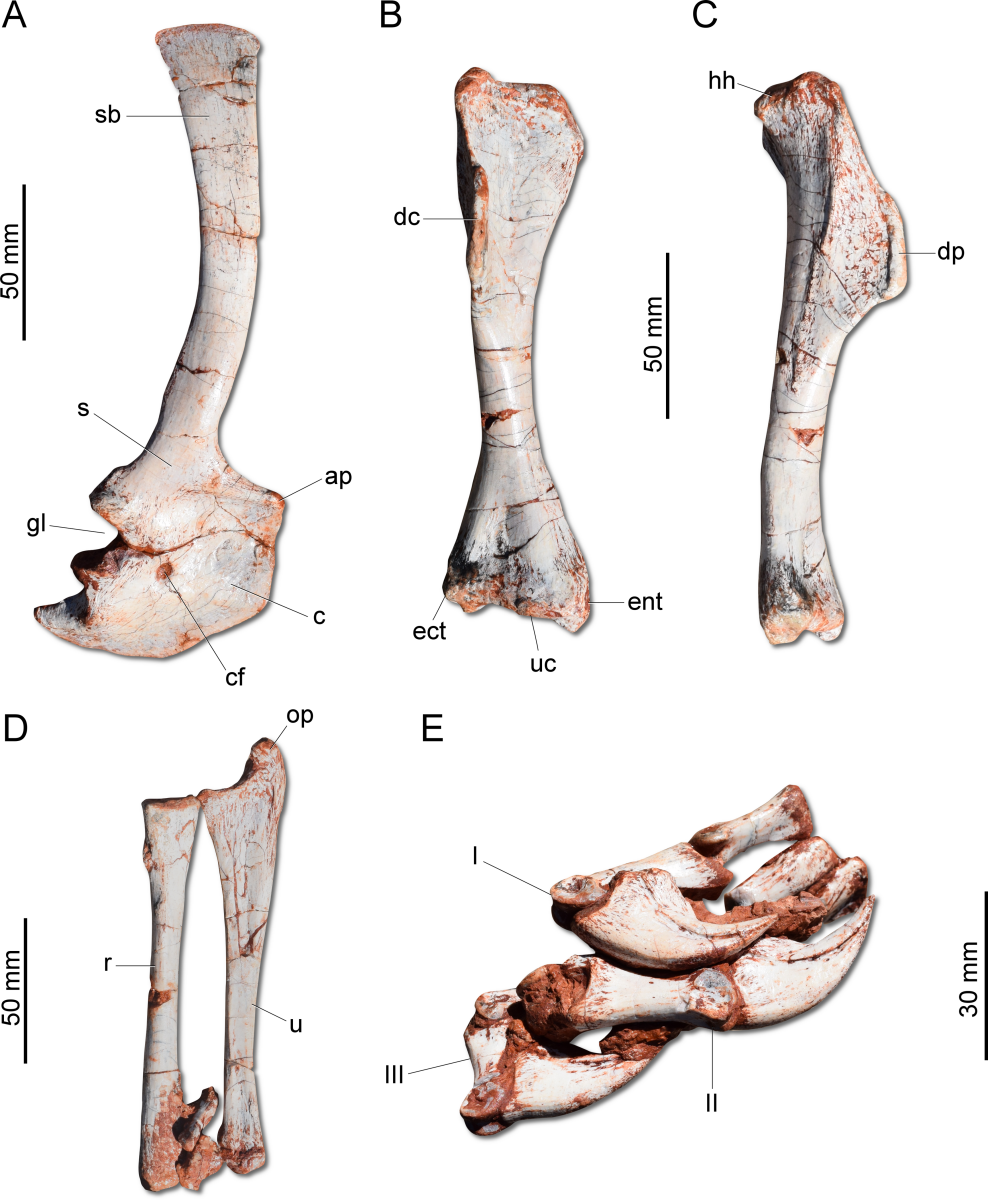
Photographs of the right shoulder girdle and forelimb of CAPPA/UFSM 0009. (A) Scapula and coidacoid in lateral view. (B) Humerus in cranial view. (C) Humerus in lateral view. (D) Radio and ulna in medial view. (E) Manus in medial view. ap, acromion process; c, coracoid; cf, coracoid foramen; dc, deltopectoral crest; ect, ectepicondyle; ent, entepicondyle; gl, glenoid; hh, humeral head; I, II, III, digits I–III; op, olecranon process; r, radius; s, scapula; sb, scapular blade; u, ulna; uc, ulnar condyle.

Unlike *H. ischigualastensis* and *San. gordilloi,* the scapula and coracoid (Fig. 6A) are not co-ossified in *G. cabreirai*. In addition, the glenoid is formed almost equally by both bones, differing from other herrerasaurids, in which the coracoid forms most of the glenoid area (Sereno, 1993; Alcober & Martinez, 2010). The distal end of the scapula expands craniocaudally, differing from *San. gordilloi* and *St. pricei* in which that part of the bone retains the same width of the midshaft. As in *San. gordilloi*, the scapular shaft is curved caudally, differing from the nearly straight blade of *H*. *ischigualastensis*. The coracoid is plate-shaped and broader craniocaudally than dorsoventrally, resembling that of *H*. *ischigualastensis* and *San. gordilloi*. The humeral shaft is nearly straight in cranial view, but sinuous in lateral view (Figs. 6B–6C). As in *H. ischigualastensis*, a prominent finger-shaped medial tuberosity is present in its proximal end, separated from the head by a deep trough. At the distal end, both ulnar and radial condyles are well developed, with prominent medial and lateral epicondyles. The ulna bears a well-developed olecranon process (Fig. 6D), as in *H. ischigualastensis* and *Saturnalia tupiniquim*. Although such a large process is also seen in other dinosaurs, it is unusual in early members of the group. The medial surface of the proximal end of the ulna is convex, resembling the condition in *H. ischigualastensis,* whereas it is slightly concave in *San*. *gordilloi*. The ulna-ulnare articulation has a strong concave-convex arrangement, similar to that of *H. ischigualastensis*, and the centrale is preserved distal to the radiale, in a configuration seemingly unique for herrerasaurids. In other dinosaurs, the centrale is located distal to either the ulnare or the intermedium (Sereno, 1993). The manus bears five digits and is elongated, more than 50% the length of the humerus. Digit and metacarpal III are longer than the others (Fig. 6E). As in *H. ischigualastensis*, manual digit IV is reduced relative to first three digits (digit and metacarpal IV measure 40 mm in proximodistal length, contrasting with 160 mm for digit and metacarpal III). There is no preserved evidence of digit V or its corresponding metacarpal.

**Figure 7:**
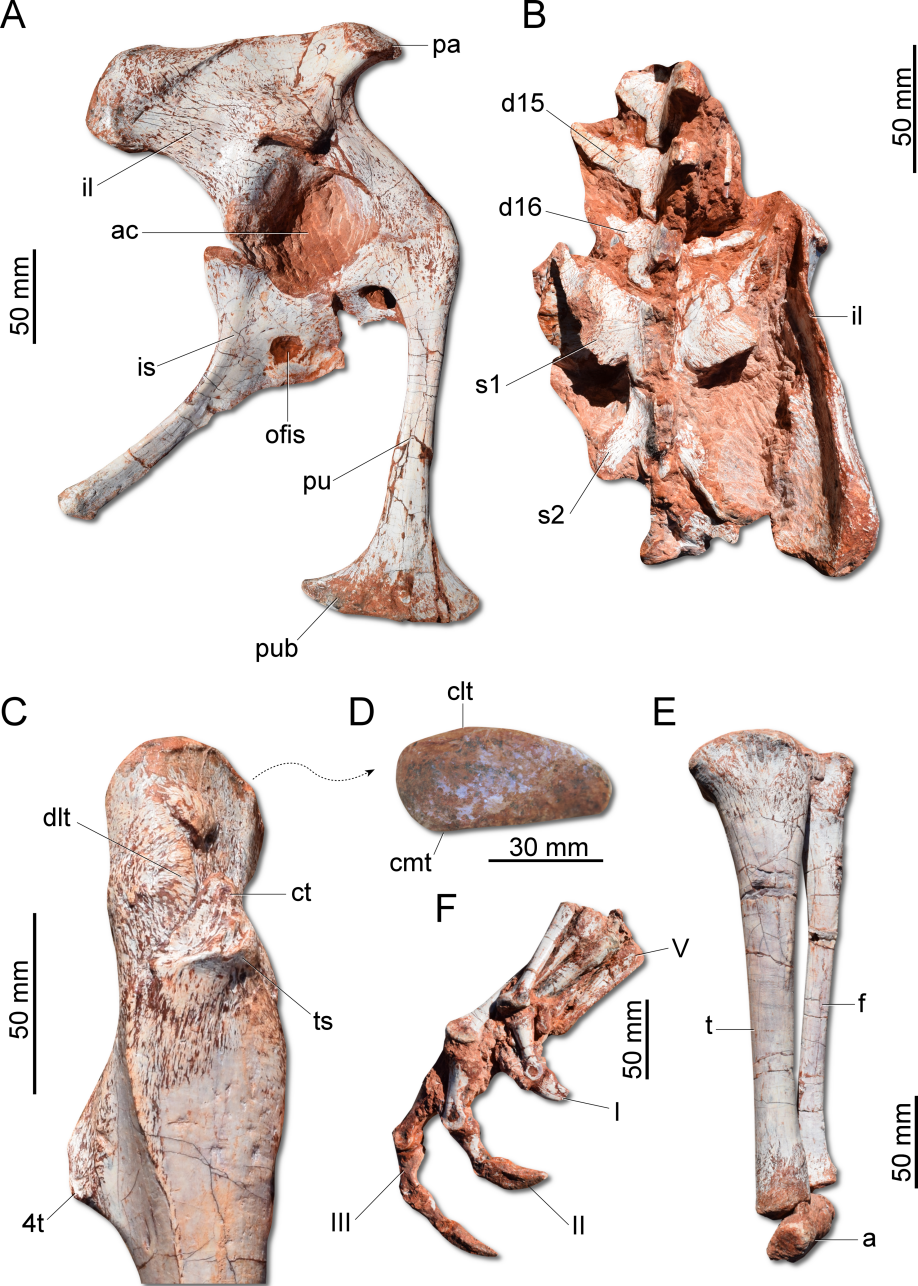
Photographs of the pelvic girdle and hindlimb of CAPPA/UFSM 0009. (A) Right pelvic girdle in lateral view. (B) Sacrum in dorsal view. (C) Proximal portion of the right femur in lateral view. (D) Right femur in proximal view. (E) Left zeugopodium in medial view. (F) Right pes in medial view. 4t, fourth trochanter; a, astragalus; ac, acetabulum; clt, craniolateral tuber; cmt, craniomedial tuber; ct, cranial trochanter; d15, d16, dorsal vertebra 15 and 16; dlt, dorsolateral trochanter; f, fibula; I, II, III, digits I to III; il, ilium; is, ischium; ofis, obturator foramen of the ischium; pa, preacetabular process; pu, pubis; pub, pubic boot; s1, s2, first and second sacral vertebrae; t, tibia; ts, trochanteric shelf; V, digit V.

The ilium (Fig. 7A) is proportionally craniocaudally short compared to the condition of other coeval non-herrerasaurid dinosaurs. In dorsal view, the cranial and caudal portions of the iliac blade are thickened, whereas the midportion of the blade is lateromedially thin, showing a concavity that faces laterally. The preacetabular process tapers cranially forming a sharp point, differing from the rounded shape observed in *H. ischigualastensis* and *St. pricei*. Though cranially projected, the preacetabular area does not surpass the cranialmost extent of the pubic peduncle. The postacetabular process is short, bearing a horizontal furrow in its ventral margin, which is similar to the condition of *H. ischigualastensis*, neither presenting the deep pockets seen in neotheropods. Indeed, this furrow is reduced in *G. cabreirai*, even if compared with those of other herrerasaurids. The stout pubic peduncle is cranioventrally directed and extends beyond the preacetabular process. The acetabulum is rounded with a laterally projected supracetabular crest in its craniodorsal margin and a well-developed antitrochanter. The shaft of the pubis is strongly bent caudally, resulting in a vertical orientation (Fig. 7A), distinct from *San. gordilloi*. The lateral surface of the pubis is sinuous as in *H. ischigualastensis* and *San. gordilloi*. Conversely, the pubic shaft of *St. pricei* is straight in cranial view. Distally, the pubis has a craniocaudal expansion as in other herrerasaurids, although *G. cabreirai* lacks the distal bevel present in *St. pricei*. This is because the caudal part of the pubic foot is craniocaudally expanded in the new species as in *H. ischigualastensis* and *San. gordilloi.* In *St. pricei* the medial portion of the distal end is proximally deflected, forming the proximal wall of the bevelled area (Bittencourt & Kellner, 2009). The ischium of *G. cabreirai* bears a large foramen in the obturator plate (Fig. 7A). This is a unique feature of the new species among herrerasaurids. However, a similar trait is observed in the lagerpetid *Ixalerpeton polesinensis* (Cabreira et al., 2016). Similar to *St. pricei*, *G. cabreirai* has a shallow excavation distal to the caudoproximal process of the ischium (caudal ischial excavation).

The femur (Fig. 7C) of the new species is sigmoid in lateral and cranial views and differs from that of other herrerasaurids in the absence of the caudomedial tuber on the proximal end (Fig. 7D). The craniomedial tuber is smooth as in *St. pricei* and less pronounced than in *H. ischigualastensis*. The proximal outline of the femur is kidney-shaped, with a shallow groove on the proximal articular surface. The proximal tip of the cranial trochanter does not form a wing-like projection and the distal portion is connected to a well-developed trochanteric shelf. The dorsolateral trochanter is rounded and extends along the anterior surface of the proximal portion of the bone. The fourth trochanter is aliform and subrectangular in lateral/medial view. It projects caudally and is located proximal to the midpoint of the femur, similar to that of *H. ischigualastensis*. The tibia (Fig. 7E) is 90% of the length of the femur, as in *H. ischigualastensis* and *San. gordilloi*, whereas in *St. pricei* the tibia is longer than the femur. Distal to the cnemial crest, the cranial margin of the tibial shaft is concave in lateral view, as in *H. ischigualastensis,* but unlike the straight margin of *San. gordilloi* and *St. pricei*. The proximal end of the fibula is slightly flattened lateromedially and the distal end is rounded, with caudomedial and caudolateral prominences, as in *H. ischigualastensis.* The pes (Fig. 7F) of the new species has three phalanges in digit V, a condition that differs from the single phalanx seen in the pedal digit V of *H. ischigualastensis*.

**Figure 8:**
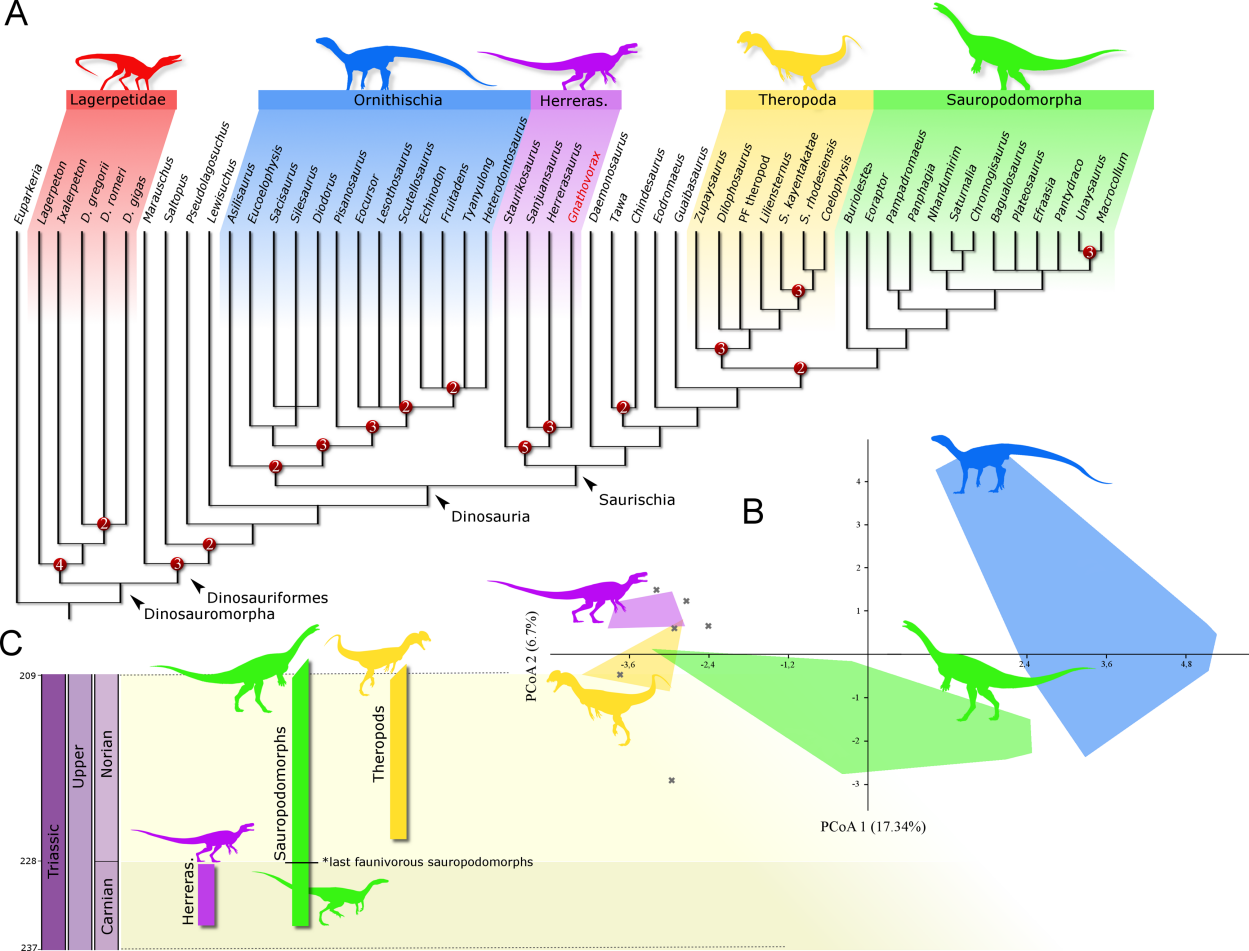
Results of the analyses. (A) Strict consensus tree depicting the phylogenetic position of *Gnathovorax cabreirai* (numbers represent Bremer support values higher than 1). (B) Bivariate plot showing the result of the morphological disparity analysis. Green convex hull corresponds to morphospace of sauropodomorphs, blue convex hull corresponds to morphospace of ornithischians, yellow convex hull corresponds to morphospace of theropods, purple convex hull corresponds to morphospace of herrerasaurids, and ‘X’, are other archosaurs. (C) Geochronological distribution of herrerasaurids, sauropodomorphs, and theropods. Note: silhouettes are composites from different sources.

**Phylogenetic position:** A phylogenetic analysis recovered 90 most parsimonious trees (MPTs) of 891 steps each, with a consistency index of 0.334 and a retention index of 0.663. In all the MPTs, *G. cabreirai* nests within Herrerasauridae (Fig. 8A), closer to the Argentinean taxa *H. ischigualastensis* and *San. gordilloi* than to the Brazilian *St. pricei*. This less inclusive clade is supported by the distal portion of the neural spine of the trunk vertebrae with a lateromedial expansion [92 (0 → 1)], a subtriangular outline of the distal end of the ischium [169 (1 → 2)], and the femur longer than the tibia [198 (0 → 1)]. Herrerasauridae nests external to the Theropoda/Sauropodomorpha dichotomy, as is also the case of some other saurischians (e.g., *Daemonosaurus chauliodus*; *Eodromaeus murphi*; *Tawa hallae*).

## Discussion

The new specimen is well supported as a member of Herrerasauridae in our phylogenetic analysis. This renders the new specimen the first herrerasaurid recorded from Brazilian strata, since the discovery of the type of *St. pricei* in 1936 (Colbert, 1970). Moreover, the unique combination of traits supports the assignation of a new taxonomic unit, increasing the diversity of the group. Additionally, the new specimen comprises the most complete and best preserved herrerasaurid dinosaur ever found. Though *H. ischigualastensis* has its skeleton substantially well sampled, this is achieved by the combination of several specimens (Novas, 1993; Sereno, 1993; Sereno & Novas, 1993), whereas *G. cabreirai* consists of a single individual that preserved its skeleton almost entirely. The lack of carbonatic concretions and only negligible compression suffered by the specimen result in an almost undistorted fossil, what also contributes to its superb preservation state. In fact, this exceptional preservation enabled us to access the morphology of the endocranial cavity, an anatomical portion poorly understood for the group. For instance, the brain endocast of *G. cabreirai* shows well-developed floccular and parafloccular lobes of the cerebellum (FFL) (Fig. 4E). This is a plesiomorphic feature among dinosaurs (Bronzati et al., 2017), documented in hindbrain endocasts of Triassic archosauromorphs of different lineages, such as the archosauriforms *Triopticus primus* and *Gracilisuchus stipanicicorum*, and the dinosaurs *Saturnalia tupiniquim* and *H. ischigualastensis* (Stocker et al., 2016; Bronzati et al., 2017). The FFL has been related to the controlling system of eye movement, neck, and head (Witmer et al., 2003; Balanoff, Bever & Ikejiri, 2010; Lautenschlager et al., 2012; Bronzati et al., 2017 but see Ferreira-Cardoso et al., 2017). In sauropodomorphs, it is hypothesized that the reduction of this structure is related to the acquisition of herbivorous feeding habits (Bronzati et al., 2017). Its retention in *G. cabreirai* is congruent with a predatory behaviour, as supported by its typically carnivorous teeth and trenchant unguals. Indeed, a morphological disparity analysis shows that herrerasaurids occupied a particular area in the morphospace of faunivorous dinosaurs (Fig. 8B), part of which overlaps with that of theropods. Also, a small portion of the morphospace area of sauropodomorphs invades that of theropods, but there is no overlap between the polygons of Carnian faunivorous sauropodomorphs, such as *Buriolestes schultzi, Saturnalia tupiniquim*, and *Eoraptor lunensis* (Cabreira et al., 2016; Bronzati, Müller & Langer, 2019), and those of herrerasaurids. Interestingly, all uncontroversial theropods are known from post-Carnian beds (*Nhandumirim waldsangae* nests herein as a saturnaliin sauropodomorph), whereas herrerasaurids and the faunivorous sauropodomorphs are Carnian in age (Fig. 8C). This suggests that the Carnian dinosaurs that adopted faunivorous diets were split between those two clades (i.e., Herrerasauridae and Sauropodomorpha) with no overlap in their ecomorphospace. Later on, however, this ecological role was taken solely by theropods, with a set of distinct body plans. Indeed, it is interesting that whereas herrerasaurids were medium to large bodied, Carnian faunivorous sauropodomorphs were significantly smaller. Conversely, Norian theropods exhibit a size variation similar to that covered by both Carnian groups together; e.g., there were small forms such as the Snyder Quarry theropod and *Herrerasaurus*-size taxa such as *Liliensternus liliensterni*. Indeed, there is no unequivocal herrerasaurid from post-Carnian rocks, whereas Norian and younger sauropodomorphs bear a set of traits related to omnivorous or herbivorous feeding behaviours (Müller, Langer & Dias-da Silva, 2018b; Müller & Garcia, 2019). Therefore, the extinction of herrerasaurids and the diet shift seen in sauropodomorphs (from faunivory to omnivory/herbivory) could both be related to the competition with theropods.

## Conclusions

The new skeleton comprises a new herrerasaurid dinosaur. This is particularly interesting because these dinosaurs are extremely rare components on Late Triassic land ecosystems (so far, only one specimen was exhumed from Brazilian beds—eight decades ago).

The superb state of preservation allowed us to access the internal anatomy of the skull through CT-scanning. Employing such approach, the endocranial soft tissues were reconstructed, revealing aspects of the neuroanatomy never explored before for such group of dinosaurs, including the recognition of a well-developed FFL, possibly related to motor control of the eye and head, which in turn may be related to predatory habit.

An ecomorphological analysis employing dental traits indicates that herrerasaurids occupy a particular area in the morphospace of faunivorous dinosaurs, which partially overlaps the area occupied by post-Carnian theropods. This indicates that herrerasaurid dinosaurs preceded the ecological role that posteriorly would be occupied by medium to large-sized theropods.

Finally, the fine preservation of the specimen renders it an important source of anatomical data on herrerasaurids and early dinosaurs as a whole. This was partially explored in this work, but additional information and the detailed anatomy of *Gnathovorax cabreirai* will be assessed and presented in future studies.

##  Supplemental Information

10.7717/peerj.7963/supp-1 Supplemental Information 1 Datasets adopted in the phylogenetic and morphological disparity analyses

10.7717/peerj.7963/supp-2 Supplemental Information 2 TNT file employed in the phylogenetic analysis

10.7717/peerj.7963/supp-3 Supplemental Information 3 3D model of the skull of CAPPA/UFSM 0009

10.7717/peerj.7963/supp-4 Supplemental Information 4 3D model of the braincase of CAPPA/UFSM 0009

10.7717/peerj.7963/supp-5 Supplemental Information 5 3D model of the endocast of CAPPA/UFSM 0009

## Additional Information and Declarations

### Data Availability

The following information was supplied regarding data availability: The Supplemental Files include the dataset adopted for the phylogenetic analysis and for the morphological disparity analysis. The specimen described in this study is stored in the collection of the Centro de Apoio à Pesquisa Paleontológica da Quarta Colônia/Universidade Federal de Santa Maria under the number: CAPPA/UFSM 0009. The 3D models are available at MorphoMuseuM: https://doi.org/10.18563/journal.m3.103 and from Pacheco et al. (2019).

### New Species Registration

The following information was supplied regarding the registration of a newly described species: Publication LSID: urn:lsid:zoobank.org:pub:B555EB12-8CEA-4043-A652-4AFE6B02EC97. *Gnathovorax* LSID: urn:lsid:zoobank.org:act:89299105-C005-46F8-A9C3-12FB93FB2002. *Gnathovorax cabreirai* LSID: urn:lsid:zoobank.org:act:500F75EF-DC9B-44B2-9EF6-765FB3374D30.
