# Supplementary material for: Gnathovorax cabreirai: a new early dinosaur and the origin and initial radiation of predatory dinosaurs
Source: PeerJ. 2019 Nov 8;7:e7963. doi: 10.7717/peerj.7963 (PMC6844243; doi:10.7717/peerj.7963)
Supplement: Supplemental Information 1 [file peerj-07-7963-s001.docx]

**Data matrix (phylogenetic analysis)**

Euparkeria capensis

00100?000000?010000000100000[01]?1011000000[01]0000000000?0001000001000000000000000000010000000?0100101000000000??01000001000000???00000100??0???0??0010000?00?0101000?000000000000010000000000000?000000[01]000000000000000000?00?000?000000000000000000010?00010000?000000

Lagerpeton chanarensis

??????????????????????????????????????????????????????????????????????????????????????????????1?00000000000???????????????????????????????????0001000?0000010000?000000000000000000100010100010?0010010110000010000100?[012]11100?01101000011000001010????020??????????

Dromomeron gregorii

????????????????????????????????????????????????????????????????????????????????????????????????????????????????????????????????????????????????????????????????????????????0000000?01010100?00101210101000000?000010??????????????????????????????????????????????

Dromomeron romeri

????????????????????????????????????????????????????????????????????????????????????????????????????????????????????????????????????????????????????????????????????????????000000010001010000?10121010110000010000100?011100?011010?????????????0?????????????????

Dromomeron gigas

????????????????????????????????????????????????????????????????????????????????????????????????????????????????????????????????????????????????????????????????????????????000000?100010100?0?101210??????????????????????????????????????????????????????????????

Ixalerpeton polesinensis

0????????????????????????????????????1??11????0????????????????????????????????0?1??0010?0???0100000000000???00????00000??????????????????????0001001?00000?0?0???0?0?0000000000000100010100010?0020010110100010000???????????????????????????????????0000001100???

Marasuchus lilloensis

???????????????????????????????????01?1?0?????????0???????????0?000010?????01?0?00000000?0000010000000000?000101000020000?????????????????????[01]0101[01]0?100001100001101100001110100010[01]1001100110?00001101000000?0100001?0010001?00?0010011000000110???0000???01000??

Saltopus elginenis

??????????????????????????????????????????????????????????????????????????????????????????????????000??????0?10??10??00?10???????????0?1??0????0?????????0???000?????01??1???????????1??1????????????1?????????????????????????00???????1?1???1?1???????????????0??

Lewisuchus admixtus

????????0000101??????011?000?0100010111111???0???????????????200000000????01001?1?000000?0?100?01????????????1011?002??0??????????????????????????????????????????????????????????????????????????????0110??100110???????????????????????????????1?000????101101??1

Pseudolagosuchus major

?????????????????????????????????????????????????????????????????????????????????????????????????????????????????????????????????????????????????????????????10000?110??????00000100?11??111110?????11?10???100?1?10?1?0???????0???????????????????????????????????

Asilisaurus kongwe

???????????????????????????????????????????????01??????????????00??111?0????0??0?1000000??10101010???10?????01?11?002??????????????????????????000?00?101???1?00??1?1?1?11??111101?01110[01]111?101?0001?0200001000100001?1000001200000???????0?????0???10????????????

Diodorus scytobrachion

??????????????????????0????????????????????????????????????????10?0111?11?????????????????????????????????????????00??00????????????????????????????????????????????????????101111?01[12]10?0111[01]0?10001????????????????????????????????????????????????1?????????????

Eucoelophysis baldwini

?????????????????????????????????????????????????????????????????????????????????????????????????????????????????????????????????????????????????????????????100101111??????111101?012110011?001?0001??11000???????????????????????????????1?1?????????????????????

Silesaurus opolensis

0?00000?0010000??0???101?110?010100110111000?01010?011001002001201111101[01]?100010011000101010101010101110010001111000000000????????????????????10002[01]01101001110010101111101110[12]111101210[01]01110011000100[12]110020001010110010?001100010????1010010?100001021?1000110?0

Sacisaurus agudoensis

????????0000101?????????0??????????????????????01????1????????12011111011?????????????????????????????????0??11??????????0?????????????????????000[12]0??101???1100??1?1?11????1[01][12]1111012100011100?1000110111002110101??????????????????????????????????102???????????

Pisanosaurus mertii

???????????0?????????????????????????????????????1??11?0101???1?11111101?????????????????????0?01????1?0??????????????????????????????????????????????????????0???????????1????????????????????0?0001?011110?0011?00?1?111?110?000[01]1????1??101???0???1?????????????

Scutellosaurus lawleri

????0????1?0??1???????????0???0????????????????1?0?1?10????2??1201111101??????00??0??0?0??0?101000???10???0001?000011000?0?????00???????0?????01??????00?????11?????1?01??11110002201310[01]011111000001102001021111010010211?000001111?????????110?1???1???????????00

Lesothosaurus diagnosticus

00000000110000100000100110000?00111010?00000101110?011001112001201111101001010?00?0100?0??001?????111?000????1000101100010????0000000??000?0000111111?000010?1100?111?0100211020022013000110111000001102001020?1101??1??1???????1?1?0??01010011??00001010111011?0?0

Eocursor parvus

0??????????????????????????????????01???0????????1?11100110????201?1110???????????0?00?1??0??0??001?1?0?0????11???01??01??????????????????????010022000000100?1???111?010?2?1110022013100111111?000011020?102011101??1??????????????????1????????0???1010???1111???

Heterodontosaurus tucki

000011001100121000001001110001?001?001100000101111?111011110001210010100011000000001000000011?1001111?000?000110001120001110001110011001100000011?221000001001100?111?101021??????2??30?0110111?0000110201???0?1????????1??????11??10000101101??1011010001110110001

Tianyulong confuciusi

?0??111?1?001?1????????????????????????????????111011?????????12???10??00????????????????????????????????????????????????????011?????10?10???????????????????????????????????????????????????????????????????0?????????????????????????????????????????????????????

Fruitadens haagarorum

????????11?0???????????????????????????????????11?0??1?????00?12?0110??00????????????0?00????????1???0?????00????????000????????????????????????????????????????????????????????0221?3000110111?0000?10201???0?11?1??1??1??????11??????????????????????????????????

Echinodon becklesii

???????????0???????????????????????????????????1110111????????12?01100000??????????????????????????????????????????????????????????????????????????????????????????????????????????????????????????????????????????????????????????????????????????????????????????

Herrerasaurus ischigualastensis

0000101?0000100000100011010001110000?00?0?0000100100000100010100000000000?1001?01?111011?0111?00101000111011101?1111[12]10101[012]10111100001001021102010120?10100011010111111120111111021011001110111?00001002100010011010111101?002000010011010101101000000010110?????00

Staurikosaurus pricei

0??????????????????????????????????????????????0010?00??0?0????0000000000?????00?011?010?0?01?001001001110111?????????????????????????????????1010120?10100011011111111?1??1111102101[01]00?111?10?0000110210001000101??????????????????????????????0???0010??????????

Sanjuansaurus gordilloi

????????0000100??????????????????????????????????????????????100000000?????00?11??1110110011100?10101011101??01111???????1???????????????????????????????????0?2011111??????1021021011001111?1000?000002000010001?10?11101?00?01??10?????????????????0?????????????

Gnathovorax cabreirai

0000101?000010000?100011?100011100001001000000?0010000?100000100000000000??0011010111010?0?11?0010000011101110111111110101???1111000010010211?001012011010001001011111112011112102101100111?111?00001002100010011010?11??1?00??000?0?11010101101000000010???1?1???0

Nhandumirim waldsangae

?????????????????????????????????????????????????????????????????????????????????????????????01??00?10?????10?????????????????????????????????000?2101110??11???????????????11110210110101111111000011??????1011111??1??????????????????1?11?0????????0?0??????????

Panphagia protos

0??????????????1?0???????????????????????0????1000100011100???[01]1000010000??????0?11110000?1011?010????110?01010?????????????????????????????????002101100??11??000???0111111??????????????1???????????02000010011010011111?00200????????0????????????1020?1??????1?

Eoraptor lunensis

0110111?0011011110110111100011100100??????100010001?000010?101[01]100000000000???1??00110000011111010101??1??0?00010121210110??100010111100002010100121011101?0110000111011[12]121???1??1011??1?1?111?00001102010010011010011111?00200001011101010111110111111111?????101

Pampadromaeus barberenai

002[12]11?100111[01]1??0?101011001?1?????????????0???00000?0???001020100001000000???????????????10111010??101?01?1?10??????0?100????????????????????100?2101??0???1?0???1?1???????11110[12]1011?011111111??00?10[12]0????0????????????????????????????????????11?10101???????11

Buriolestes schultzi

0?210111001100111?1101?110101111010010010??00110001?001110010200000000?00101011011111000001011101001101101000101?111120110????0???01??????????100111011001001?0110111?1111111111021011001111111?00001102010010011?10???110?????000100???001111111010001111100111101

Saturnalia tupiniquim

1?????????????????11?1?????????????010???0?????00?1???????????11000010000?????10?1?110000010101010011011010101010121201111????????????????????100[01]21011001?1110100111011[12]1111111021011011111111?00001002010011011010011101?002[01]00010?1101011111110???1021?1?0101???

Chromogisaurus novasi

???????????????????????????????????????????????????????????????????????????????????????????????????????????1?????????????1??????????????????????012101?0?1??????????????????????????????1????111????1?0201001101101???????????????????????????????????020??????????

Bagualosaurus agudoensis

1?2[12]001?0011001????1???1?1??1?1????????????100?0011100?11?0102120000100??0????????????????1?111?1001?0110??1??????????????????????????????????10012101?0??01??0???111?????????????1011??1???111000??1002?0??1100111?????????????????????0?11111110???1?10??????????

Pantydraco caducus

????101??0?????????????0??????????101??????0??10011??0?????10?12?0001001001????01111?000??10???????????????00??00??11?????????????????????????0111???1?1?0010?0???????11[12]1???????????????????1??00001??200???00?1?1?0???????????????????0010111210???100111?0110000

Efraasia minor

1?2110100020101?111101???1?00????100100001?1??10011101101?01?2120?00110000????10?111100???10101010?0?011010101010121201110011?0101111?1101[12]000001022011001?111000011101121111111021011000111111000001002000011011010011211?????00?1001100010111200???101011?01100??

Plateosaurus engelhardti

11211010002010111111010011[01]00?0111001000011100100111011010020212010011100010111011111010011011101001101101010101002120111001100101111111000000001022011101?111010011101121[12]111[01]102101100011111100000000200001[01]111010011201?0020000101110001011020010010101101110100

Unaysaurus tolentinoi

?122101?0021111????????????????11??????????1?0100?1????????10212010011?000?0?1????????????1011101??????????101010?212?0110????????11????????????????????????????????????????????????????????????????????????20011?1011??01???20??????????????????0?????????????????

Macrocollum itaquii

11221011002111111?11010111100?11110???????110010011?0?10100102120100110000???110111110000110111010?????1010101010121200?10??1?01?1111?????????000021011001?111000011101121111101021011000110111?00001002000020011[01]10111101?0?200001011100011111100100101011????????

Chindesaurus briansmalli

?????????????????????????????????????????????????????????????????????????????????????????????????0??10?1????0????????????????????????????????????1[12][12]??1??????1??????????????1011021011001110110?0?001???1?0120001[01]10111111?112[01]0???????????????????????????????????

Tawa hallae

012211?00020100???1000?001010?0100?0100000100010000000?00?001000000000?001????11112111111?10111?10?000??0?0?001??111[12]10100000101110001001011?1?010120?101??011020?1011?1??2?1021022111110111100100001102110120001010111111?11210001101100011111010111000011?111100?

Eodromaeus murphi

0?1??0??0011010???????1??0????1?00????????0????0000100???0?1?000000000000?0?0011?1???110?0?01?1110?0??????010111?11121011111010?1?0100?110[12]10010102101101001[12]1011?10?111??11???1?21?11001?1?????00001012210010011110?111?0?002000011?????0???????0???0020???0101???

Coelophysis bauri

012212?100210011100101111[01]0110100111101000010110000100?1000112000000001001111111112111101?101011111110010100110111?1?10110211111110111111121?1210022111101?121021?10111111212?0002211200111110010000[01]1122200????1110110111?0020110110010111101101010001011110????01

Liliensternus liliensterni

?????????0?1???????????????1??1????????????????0000?00????????000?00?0?00?????11?121?110?11010111?11100?0?????????11110?10??????????1?????????210022011111?121021?10111111212111022112110111110?00001002220020111110010111?0020110?1????1?1101???0???0121??????????

Syntarsus rhodesiensis

01221211?011000110010?101?????10011111??00?1???0000000?????112000000?010011???11112111101?101?1111111001010011111111210?11211111?11111011121?1210122111101?1?100??1011111121211102211[12]01[01]111100?00000112220020111010010111?0020111?1?010111101101011?0111?1101012??

Syntarsus kayentakatae

01221211?021010?101101101?10101110???00?0?010010000000?1000112000000?00001????11112111101?101?111111100101??110111???1????????????????????11?1??????1??1???1?1021?101111??2?211102?1120011111001000011122200??????10?1?????????111?1?0??111101???01000101111?1????0

Zupaysaurus rugeiri

?????2???011010?101101011101100011???00?????01100?0100?1000??2000000?0000??1???????????????????????????????????1????????????????????????????????11???????????????????????????????????????????????0001???????21111110010101?0000010?1??????????????1100???1???????00

Daemonosaurus chauliodus

?00011???010100?0010???11001001001???00???111010010000?0000000?00000?0000??????10?????????????????????????????????????????????????????????????????????????????????????????????????????????????????????????????????????????????????????????????????0110???1????????0

Petrified forest theropod

????????????????????????????????????????????????????????????????????????????????????????????????????????????????????????????????????????????????00221111?1??11021?1?111?112?211102?112101111110??0001?0212002111111001010??0??0110?100101?11?1????????111??????????

Dilophosaurus wetherelli

012[12]?2?10010010???11?0101101100101?1111001110010010000110?01110000000?000111110111111210?110101110111011010011011121[12]10110???111111101100121?1210022011101?121021?1111111121211102211210[01]111110100000002120021111110010101?00210111100101111011010011012111101?0??0

Guaibasaurus candelariensis

??????????????????????????????????????????????????????????????????????????????????????????1?1111100001110?0???0??111?10110???1111111100111?1??100121[01]1101??11100?0111[01]11?111??????1?11?00?1?11[01]10000?012100020011110111101?01200011011100011111110????020??????????

**Data matrix (morphological disparity analysis)**

Euparkeria capensis 00100000000000000

Marasuchus lilloensis ???0?000010?????0

Lewisuchus admixtus ??200000000????00

Asilisaurus kongwe ????00??111?0???1

Diodorus scytobrachion ????10?0111?11??1

Silesaurus opolensis 20012011111011?11

Sacisaurus agudoensis ???12011111011??1

Pisanosaurus mertii ???1?11111101???1

Scutellosaurus lawleri 2??1201111101???1

Lesothosaurus diagnosticus 20012011111010011

Eocursor parvus ????201?1110????1

Heterodontosaurus tucki 00012100101000111

Herrerasaurus ischigualastensis 10100000000000?10

Staurikosaurus pricei ????0000000000??0

Sanjuansaurus gordilloi ??100000000?????0

Panphagia protos ???11000010000??1

Eoraptor lunensis 10111000000000001

Pampadromaeus barberenai 10201000010000001

Buriolestes schultzi 10200000000?00100

Saturnalia tupiniquim ???11000010000??1

Pantydraco caducus 10?12?00010010011

Efraasia minor 1?2120?00110000?1

Plateosaurus engelhardti 20212010011100011

Tawa hallae 01000000000?001?0

Eodromaeus murphi 1?000000000000?00

Coelophysis bauri 11200000000100110

Liliensternus liliensterni ???000?00?0?00??0

Syntarsus rhodesiensis 112000000?0100110

Syntarsus kayentakatae 112000000?00001?0

Zupaysaurus rugeiri ??2000000?0000??0

Daemonosaurus chauliodus 000?00000?0000??0

Dilophosaurus wetherelli 1110000000?000110

Gnathovorax cabreirai 00100000000000??0

Unaysaurus tolentinoi 10212010011?000??

Macrocollum itaquii 102120100110000?1
